# Acupuncture for persistent allergic rhinitis: a multi-centre, randomised, controlled trial protocol

**DOI:** 10.1186/1745-6215-10-54

**Published:** 2009-07-14

**Authors:** Jong-In Kim, Myeong Soo Lee, So-Young Jung, Jun-Yong Choi, Sanghoon Lee, Jeong-Min Ko, Hong Zhao, Jiping Zhao, Ae-Ran Kim, Mi-Suk Shin, Kyung-Won Kang, Hee-Jung Jung, Tae-Hun Kim, Baoyan Liu, Sun-Mi Choi

**Affiliations:** 1 Medical Research Centre, Korea Institute of Oriental Medicine, Daejeon, Republic of Korea; 2 Department of Acupuncture & Moxibustion, College of Korean Medicine, Kyung Hee University, Seoul, Republic of Korea; 3 Department of Acupuncture & Moxibustion, Guang'anmen hospital, China Academy of Chinese Medical Science, Beijing, PR China; 4 Department of Acupuncture & Moxibustion, Dongzhimen hospital, Beijing University of Chinese Medicine, Beijing, PR China; 5 Department of Clinical Research Centre, China Academy of Chinese Medical Sciences, Beijing, PR China

## Abstract

**Background:**

Allergic rhinitis is one of the most common health complaints worldwide. Complementary and alternative medical approaches have been employed to relieve allergic rhinitis symptoms and to avoid the side effects of conventional medication. Acupuncture has been widely used to treat patients with allergic rhinitis, but the available evidence of its effectiveness is insufficient. Our objective is to evaluate the effectiveness of acupuncture in patients in Korea and China with persistent allergic rhinitis compared to sham acupuncture treatment or waitlist control.

**Methods:**

This study consists of a multi-centre (two centres in Korea and two centres in China), randomised, controlled trial with three parallel arms (active acupuncture, sham acupuncture, and waitlist group). The active acupuncture and sham acupuncture groups will receive real or sham acupuncture treatment, respectively, three times per week for a total of 12 sessions over four weeks. Post-treatment follow-up will be performed a month later to complement these 12 acupuncture sessions. Participants in the waitlist group will not receive real or sham acupuncture treatments during this period but will only be required to keep recording their symptoms in a daily diary. After four weeks, the same treatment given to the active acupuncture group will be provided to the waitlist group.

**Discussion:**

This trial will provide evidence for the effectiveness of acupuncture as a treatment for persistent allergic rhinitis. The primary outcome between groups is a change in the self-reported total nasal symptom score (i.e., nasal obstruction, rhinorrhea, sneezing, and itching) from baseline at the fourth week. Secondary outcome measures include the Rhinitis Quality of Life Questionnaire score and total non-nasal symptom score (i.e., headache, itching, pain, eye-dropping). The quantity of conventional relief medication used during the follow-up period is another secondary outcome measure.

**Trial registration:**

Current Controlled Trials ISRCTN90807007

## Background

Allergic rhinitis (AR) is a highly prevalent major chronic respiratory disease. It significantly impacts quality of life (QoL) and creates an economic burden. AR can be classified as intermittent or persistent allergic rhinitis (PER), as described by the recent 'Allergic Rhinitis and its Impact on Asthma (ARIA)' guidelines [1]. The conventional treatment of AR symptoms, such as nasal obstruction, rhinorrhea, sneezing, and itching, includes the use of intranasal corticosteroids, oral anti-histamines with or without decongestants, immunotherapy, and education [2]. Substantial numbers of patients with AR are not satisfied with conventional medical treatment and repeatedly experience side effects. As a result, a large number of patients with AR are turning to complementary and alternative treatments [3,4].

Acupuncture not only produces an anti-nociceptive effect, but also has anti-inflammatory or immunomodulatory effects against chronic inflammatory conditions in humans [5]. The anti-inflammatory action of acupuncture is thought to be mediated by neural immune reflexes [6,7], i.e., the cholinergic anti-inflammatory pathway of the central nervous system [8]. Although firm evidence has not been established, acupuncture has been used by patients with AR. The results of our systematic review of six randomised controlled trials (RCTs) on acupuncture for PER revealed that acupuncture had a favourable effect on AR symptom scores compared to placebo acupuncture (standardised mean difference: 0.45, 95% CI: 0.13 – 0.78) [9]. However, the results of our review on the efficacy of acupuncture in the treatment of AR were inconclusive, consistent with other studies [10,11]. This may be because all relevant RCTs were limited by methodological defects, including inappropriate sample size, inappropriate placebo treatment, missing information, and lack of clarity about the quality of the acupuncture practitioners [12,13]. A deficiency is evident even in a recent, high-quality randomised controlled trial [14] in which acupuncture was used for PER in an eight-week treatment and 12-week follow-up period. The results showed an improvement in the cumulative seven-day scores between the active and sham acupuncture groups. In this study, Xue *et al. *used a shorter needle in the placebo procedure compared to the active acupuncture group. This suggests that their strategy may be flawed with respect to patient blinding. Furthermore, most RCTs for AR allowed the concomitant use of pharmacological medication for symptomatic relief of AR, or acupuncture was compared to conventional medication and not to placebo needle treatment. Success of the blinding procedure has not been assessed in RCTs, apart from one trial [15]. Consequently, the difference in outcomes between the two groups could be unrelated to the effect of acupuncture itself, and may be influenced by the use of over-the-counter (OTC) or conventional medications that yield symptomatic relief. Thus, disallowance of concomitant medications is needed in a randomised controlled trial for PER in order to detect the specific effects of acupuncture.

With these constraints, we planned a rigorous multi-centre randomised controlled trial with a large sample size.

## Method/design

### Objective

The aim of this study is to investigate the effectiveness of acupuncture compared with sham acupuncture or waitlist in patients with moderate to severe PER in Korea and China.

### Primary outcome

Primary outcome will be the change in the weekly average of total nasal symptom score (TNSS) recorded in participants' diaries between the one-week of run-in period and the 4P^th ^week of the study. This scoring system was previously validated [14,16].

### Secondary outcomes

Secondary outcome measures include the Rhinitis Quality of Life Questionnaire (RQLQ) [17] score and a total non-nasal symptom score (i.e., headache, itching, pain, eye-dropping) [14] after the 12-session/four-week acupuncture treatment period. The quantity of conventional relief medication used during follow-up period (four weeks) will also be recorded.

### Design

**Figure 1:**
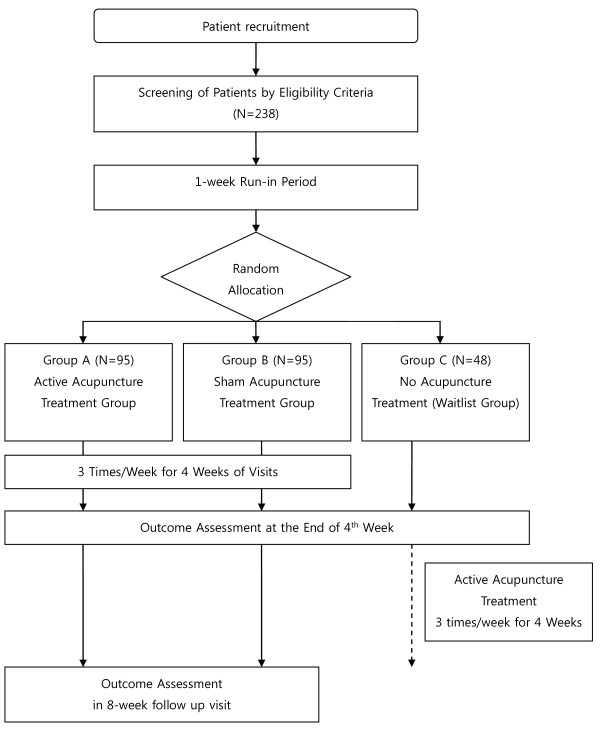
**Study Flow Chart**.

A multi-centre (two centres in Korea and two in China), randomised, subject- and assessor-blinded, sham acupuncture controlled trial will be conducted at the Department of Acupuncture and Moxibustion, Kyung-Hee University Medical Centre in Seoul, the Acupuncture and Moxibustion Research Centre of the Korean Institute of Oriental Medicine (KIOM) in Daejeon, the Acupuncture and Moxibustion Clinic of Guang'anmen Hospital, and Dongzhimen Hospital at Beijing. The study will be sequentially conducted as follows: a run-in period of one week prior to randomisation, a treatment period of four weeks (three sessions per week), and a follow-up period of four weeks. The total study period will be nine weeks. At the end of the run-in period, participants will be randomised to the active acupuncture group, the sham acupuncture group, or the waitlist group by a computer-generated random number list (Figure 1).

### Eligibility

#### Inclusion criteria

Eligible participants will be those previously diagnosed with moderate to severe PER, according to the ARIA criteria. Patients will be required to complete the baseline AR diary. Written informed consent will be obtained from each participant. Participants ≥ 18 years of age will be recruited from each centre via local newspaper advertisements and posted notices. Since the research involves moderate to severe PER or severe PER [1], the inclusion criteria will include greater than four days/week and greater than four consecutive weeks of symptoms and one or more of the following conditions: sleep disturbance, impairment of daily activities such as sports and/or leisure, impairment of school or work, or troublesome symptoms. All participants will have to show at least one positive result on an allergic skin prick reaction test. A standard panel of antigens will be used, i.e., positive (histamine) and negative (saline) controls, *Dermatophagoides farinae, Dermatophagoides pteronyssinus*, dog fur, cat fur, short ragweed, Orchard grass, Bermuda grass, Mugwort, Hazel, Alternaria tenuis, *Aspergillus fumigatus*, Candida albicans, *Cladosporium herbarum*, Alder, Beech, and cockroach (*Blatella germanica*) (Allergopharma, Germany).

Participants will be instructed to stop PER symptomatic relief medication during the run-in and treatment periods and will be provided the usual care instruction for AR. During the follow-up period, they will be allowed to start relief medication, if required.

#### Exclusion criteria

Participants will be excluded if they suffer from serious medical conditions, such as uncontrolled hypertension, diabetes mellitus requiring insulin injection, past or current malignant tumour, severe dyslipidaemia, liver or kidney dysfunction, anaemia, active pulmonary tuberculosis, or other infectious or systemic diseases inappropriate for treatment with acupuncture.

Participants will not be eligible if they have congenital nasal abnormalities, sinusitis, active asthma, a history of nose surgery, received systemically administered corticosteroids within six months prior to enrolment, or received alternative and complementary treatment, (i.e., acupuncture or herbal medication) for AR within the previous six months. Typical oral and nasal H1 blockers, intranasal/oral corticosteroids, or nasal anti-cholinergic medication will be stopped at least one week before enrolment. OTC drugs will be allowed for managing episodic colds, headaches, dyspepsia, etc. However, participants will be asked to disclose all medication use prior to enrolment. OTC drugs containing an H1 anti-histamine or anti-cholinergic component will not be allowed, since they may directly affect nasal symptoms. We will record the drugs taken by each participant at every visit, and participants will be requested to notify us of any change to their medication/supplement regimen. Additional acupuncture treatments, herbal prescriptions, or therapeutic intervention by other clinicians will not be allowed during the treatment period. Participants in the waitlist group will not receive acupuncture or sham acupuncture treatments during the study. After four weeks, if participants elect to try the acupuncture treatment, the real acupuncture treatment will be provided.

### Acupuncture treatment protocol

#### Acupuncture intervention and sham acupuncture

Acupuncture will be performed by certified acupuncture medical doctors at four centres. Qualified specialists of acupuncture in traditional Korean or Chinese medicine with at least three years of clinical experience will perform the acupuncture in this study. All treatment regimens will be standardised between Korean and Chinese practitioners *via *video and international workshops. Participants will be randomly assigned to the active acupuncture group, the sham acupuncture group, or the waitlist group. The active acupuncture and sham acupuncture group participants will receive real and sham acupuncture treatments respectively, three times/week for a total of 12 sessions over four weeks. A 0.20 mm (diameter) × 30 mm (length) disposable needle (Dongbang Acupuncture Inc, Korea) will be used. For the active acupuncture group, 10 acupuncture points (bilateral LI4, LI20, ST2, and ST36, unilateral EX-1 and GV23) according to 'WHO Standard Acupuncture Point Location' [18] were selected for use based on an expert discussion held in Beijing in December, 2008. These acupuncture points were selected according to traditional Chinese medicine (TCM) theory, which is quite different from that of conventional medicine. Briefly, the basic therapeutic strategies of acupuncture, based on a meridian system, is to correct the body's core imbalances in order to disperse blocked *Qi *and blood in treating disease [19]. According to the TCM theory, when an acupuncture point is stimulated, treatment effects tend to occur in specific parts of the body along the meridian that contains this specific acupuncture point. There are two categories of acupuncture points used to effectively regulate *Qi *and blood for syndromes: local points and distal points. In establishing an acupuncture strategy for this study, we focused on the imbalance of the Large Intestine and/or Stomach meridian and/or Governor Vessel due to an invasion of pathogenic agents, such as cold, wind, and damp, as the key pathogenesis of PER. These three key meridians associated with PER flow together through the nose, and are therefore closely connected with respiratory function. In order to disperse pathologic *Qi *and blood from these meridians and improve the function of the nose and sinuses, the local or adjacent points, including LI20 in the Large intestine meridian, ST2 in the Stomach meridian, GV23 in the Governor vessel and EX1, were selected. The remote points, including LI4 and ST36, were selected to correct the imbalance of the relevant meridians and organs. LI4 was selected for lung *Qi *deficiency syndrome, and ST36 for spleen *Qi *deficiency syndrome. These two syndromes represent the pathologic basis of PER in TCM theory.

As for treatment sessions, previous high quality trials [14,16] used a total of 16 acupuncture sessions, twice a week for eight weeks. But those trials allowed the concomitant use of conventional medication for symptomatic relief of AR, so interpretation of the efficacy of acupuncture is limited. In contrast to those studies, we added one additional session of acupuncture treatment per week (i.e., three times/week) without allowing PER symptom relievers; this addition was thought to compensate for the reduced effect of not using conventional medication. As with treatment duration, we considered four weeks to be a sufficient period of time to obtain clinically relevant improvement based on clinical experiences with PER patients. The needles will be inserted to a depth of 7–30 mm, depending on the points selected. In order to achieve *de-qi *sensation for real acupuncture treatment, the needles will be manually manipulated and maintained for 20 minutes, with manual stimulations at the start and just before the withdrawal of needles. Additional interventions, such as infrared irradiation or electronic stimulation, will not be allowed during acupuncture treatment. For real acupuncture treatment, needles will be inserted into the aforementioned acupuncture points at an appropriate insertion angle and direction (Table 1). For example, at EX1, the needle will be inserted to a depth of 20 to 30 mm in an oblique direction towards the nose with respect to the skin. Each needle will be rotated until the participant and doctor feel de-qi sensations. For the sham acupuncture treatment, the needles (the same type used for the active acupuncture treatment) will be inserted at non-acupuncture point sites, 1 – 1.5 cm from the acupuncture points. However, they will be inserted to a depth of 3 – 5 mm perpendicularly to the skin, using a hollow pool and a shallow needling technique to avoid de-qi. The needle will then be rotated once in order to preserve patient blinding. The duration of this procedure will be the same as the real acupuncture treatment.

**Table 1:** Acupuncture points and needling procedure for real and sham acupuncture.

Real Point	Angle and Direction	Depth (mm)	Sham point
LI20 (Both)	Obliquely along the nasolabial sulcus towards the root of nose with respect to the skin	20–30	Lateral 1.5 cm and Downward 1 cm to LI20.

ST2 (Both)	Transversely, upward to the centre of the pupil with respect to the skin	7–10	Lateral 1.5 cm to ST2

EX1 (unilateral)	Transversely, downward to the nose	20–30	Upward 1.5 cm, Rt. Lateral 1 cm to EX1

GV23 (unilateral)	Transversely, downward to the forehead with respect to the skin	20–30	Rt. Lateral 1 cm to GV23

LI4 (Both)	Perpendicular to the skin	20–30	Posterior to the web margin between the first and second phalanges

ST36 (Both)	Perpendicular to the skin	20–30	Medial 1.5 cm to ST36

In the sham acupuncture procedure, the needle will be inserted at each sham point to a depth of 3–5 mm, perpendicular to the skin, using a needle tube with a one-time rotation

Needle sites in both active and sham acupuncture groups will be swabbed with 2% boric acid before insertion. On withdrawal of the needle, dry sterilised cotton balls will be firmly applied to the insertion points. Blood pressure will be measured before and after acupuncture treatment. Before acupuncture, each participant will be evaluated by an assessor blinded to participants' random allocation, and their symptoms and total non-nasal symptom scores will be recorded. The level of any discomfort in the acupuncture or sham acupuncture will also be monitored.

#### Outcome measures

After screening, participants who satisfied the entry requirements will begin the run-in period. During this period, participants will document the four nasal symptoms on a diary card once per day. These scores for nasal obstruction, rhinorrhea, sneezing, and itching will be summed to yield the TNSS. Each symptom is graded on a five-point scale (0 = no symptom; 1 = mild; 2 = moderate; 3 = severe; 4 = very severe). Weekly averages of TNSS will be calculated and compared among groups throughout the study period. In addition, TNSS will be reviewed by an assessor blinded to the participants' treatment group at each visit. At the end of the run-in period, the RQLQ scores will be examined in the Korean and Mandarin Chinese versions of the questionnaire [20]. RQLQ measures the influence of AR on QoL and consists of 28 items in seven domains: sleep, non-hay fever symptoms, practical problems, nose symptoms, eye symptoms, activity, and emotional function in everyday life, all based on a sum score (range: 0 – 6). The RQLQ score will be blindly assessed three times: baseline, at the end of treatment, and at the end of the follow-up period. Participants in the active acupuncture group and sham acupuncture group will record their use of PER relief medications daily during the follow-up period.

We will estimate the expectation of participants using the credibility and expectancy questionnaire to determine whether expectations affected outcomes [21]. Blinding will be assessed by asking participants what arm they thought they were in (active or sham acupuncture group, or "don't know") at the end of trial, and why they believed they were in that arm. Adverse response of acupuncture will be recorded.

### Statistical methods

#### Analysis

Analysis will be performed for each of the following groups: 1) an intention-to-treat population consisting of all randomised participants with at least one measurable outcome report following acupuncture treatment (any missing data will be replaced with the last observation values); 2) a protocol-specific population including only participants without major protocol deviations. All data will be analysed descriptively. All main analyses will be based on the intention-to-treat population. For primary and secondary outcome measures, analysis of covariance (ANCOVA) using the differences in the change of outcome variable scores as the dependent variable, baseline outcome variable scores as the covariate, and allocated group and centres as the fixed factors will be performed. To compare the active acupuncture group with the sham acupuncture group and with the waitlist group, a single ANCOVA using dummy variables for the sham and waitlist group (thus coded 0, 0; 1, 0; 0, 1 for the active acupuncture group, sham acupuncture group, and waitlist group, respectively) will be conducted. All adverse events reported during the study will be included in the case report forms; the incidence of adverse events will be calculated. The percentage of subjects with adverse events in each group will be calculated and compared using the chi-squared test or Fisher's exact test.

Statistical analyses will be performed using the SAS statistical package program (ver. 9.1.3, SAS Institute Inc, Cary, NC), and the level of significance will be established at α = 0.05.

#### Sample size

For power analysis and sample size calculation, we conducted a pilot study with a sample size of 19 (9 in the active acupuncture arm and 10 in the sham acupuncture group). In this study, the pooled SD of TNSS was 4.74 and the mean difference of TNSS change between the active and sham acupuncture group was 2.53. This difference was larger than that of recent successful acupuncture RCT on PER, reported as 1.8 [14]. TNSS reduction in the active acupuncture group in our pilot study was 4.12, higher than the minimal clinically important differences (i.e. 2.2) [22]. Therefore, the result of our pilot study showed the potential of acupuncture treatment and supported the need for the present trial. The SD of the pilot study was adjusted for better application to the true population. In order to achieve this adjustment, we multiplied the SD from the pilot data by a correction factor of 1.148. This adjustment provides a 75% probability that the resulting estimate of SD will be at least as large as the true population SD [23]. For the sample size calculation, we established a sample size (N) for an independent t test using the adjusted SD, and the difference in TNSS change between the active and sham acupuncture group with a power of 80% and alpha value of 2.5% (two-tailed). We then estimated a Pearson correlation value (R) of 0.76 between baseline and the change of TNSS in our pilot study. Finally, we multiplied N by (1-R^2^) to yield the sample size needed for ANCOVA analysis [24]. The results indicated that the number of subjects in the active acupuncture group and in the sham acupuncture group was 38. If we compare the change in TNSS for the active acupuncture group with that of the waitlist group, assuming that pooled SD and R are similar to our pilot study, the differences between groups will be larger, and thus 38 subjects in the waitlist group size will be enough to detect significant differences in the change in TNSS for the active acupuncture group. We applied a 2:2:1 ratio to the active acupuncture group, sham acupuncture group, and waitlist group, respectively, and we expect that this allocation ratio will raise the statistical power. We plan to enrol 95, 95, and 48 participants in the active acupuncture group, sham acupuncture group, and waitlisted group, respectively, allowing for a 20% withdrawal rate.

#### Data handling

Investigators will enter the collected data into case report forms; unclear or out of range entries and omissions will be recorded on data query forms, which will be returned to the investigational site for resolution. The data from all centres will be pooled and summarised according to demographic baseline characteristics, effectiveness, and safety observations.

#### Data and safety monitoring

Regular monitoring will be conducted for quality control. Investigators will also convene regularly to discuss practical issues that may be encountered, such as adjusting the recruitment capacity within each centre, dealing with serious adverse events, revising the protocol, as well as any other important issues raised by the investigators or participants. The assessment of safety will be based mainly on the frequency of adverse events, as well as particularly serious adverse events. Information regarding adverse events will be summarised by presenting the number and percentage of participants that experienced adverse events, with the information also categorized according to the body region affected. Any other collected information (e.g., severity or relevance to acupuncture treatments) will be included in the data and safety monitoring reports.

#### Randomisation and allocation

Randomisation will be performed by researchers who are not in direct contact with the participants. Participants and assessors will be blinded to the treatment allocation. The acupuncture practitioners cannot be blinded due to the nature of the intervention, but they will not be permitted to communicate with the participants or assessors regarding treatment procedures and responses.

Ultimately, 238 participants will be randomised into the three groups at four centres. The number of subjects planned for each location will be: clinical research centre of KIOM – 70, Kyung-Hee hospital – 68, Dongzhimen hospital – 50, Guang'anmen hospital – 50. Random number generation with block randomisation will be carried out by a computer statistics software package (Version 9.1.3; SAS Institute Inc, Cary, NC) for the central control. The ratio of random allocation for the acupuncture group, sham acupuncture group, and waitlist group will be 2, 2, and 1, respectively, within each centre. The exact block size will be concealed from personnel who will contact participants directly. Separate randomisation tables will be delivered to each centre. Tables will be concealed from anyone involved in the study except for personnel involved in the random assignment.

Just before randomisation, participants will be informed that they will be assigned to one of three groups, including two that have the potential to reduce PER symptoms and a waitlist for treatment. We will not inform the participants in the active and sham groups about which treatment they receive until the end of the study. To eliminate observation bias, assessors will be blinded prior to analysis of the data.

A sealed numbered envelope for each participant will be given to researchers involved in random allocation, to be opened at each participant's randomisation visit (Visit 2). The serial numbers of sealed and open envelopes and their matching registered participants will be checked and recorded in logs just before and immediately after opening each envelope by independent personnel not in charge of opening envelopes. Any error or inappropriateness with random allocation in each centre will be reported to the central coordination centre (KIOM) immediately, and a new randomisation table will be generated beginning from the problematic serial number and applied to the participants thereafter.

### Ethics

Written consent will be obtained from each participant. This study was approved by all relevant local ethics review boards: Kyung-Hee University Medical Centre, Daejeon Oriental Hospital in Korea and Dongzhimen hospital in China.

## Abbreviations

ANCOVA: analysis of co-variance; AR: allergic rhinitis; ARIA: allergic rhinitis and its impact on asthma; CACMS: China Academy of Chinese Medical Sciences; KIOM: Korea Institute of Oriental Medicine; OTC: over-the-counter; PER: persistent allergic rhinitis; QoL: quality of life; RCTs: randomised controlled trials; RQLQ: rhinitis quality of life questionnaire; TCM: traditional Chinese medicine; TNSS: total nasal symptom score.

## Competing interests

The authors declare that they have no competing interests.

## Authors' contributions

SMC obtained funding for the research project. JIK and MSL drafted the protocol, and JIK wrote the final manuscript. SYJ, SL, JMK, JYC, HZ, JZ, ARK, and MSS, JHJ and THK contributed to the research design and made critical revisions. KWK was responsible for the statistical design of the trial and wrote portions of the statistical methods, data handling, and monitoring sections. BL and SMC are the representatives of CACMS and KIOM, respectively, and participated in the trial design as co-principal investigators. All authors read and approved the final manuscript.
